# Crystallography and Its Impact on Carbonic Anhydrase Research

**DOI:** 10.1155/2018/9419521

**Published:** 2018-09-13

**Authors:** Carrie L. Lomelino, Jacob T. Andring, Robert McKenna

**Affiliations:** University of Florida College of Medicine, Department of Biochemistry and Molecular Biology, Gainesville, FL 32610, USA

## Abstract

X-ray and neutron crystallography are powerful techniques utilized to study the structures of biomolecules. Visualization of enzymes in complex with substrate/product and the capture of intermediate states can be related to activity to facilitate understanding of the catalytic mechanism. Subsequent analysis of small molecule binding within the enzyme active site provides insight into mechanisms of inhibition, supporting the design of novel inhibitors using a structure-guided approach. The first X-ray crystal structures were determined for small, ubiquitous enzymes such as carbonic anhydrase (CA). CAs are a family of zinc metalloenzymes that catalyze the hydration of CO_2_, producing HCO_3_^−^ and a proton. The CA structure and ping-pong mechanism have been extensively studied and are well understood. Though the function of CA plays an important role in a variety of physiological functions, CA has also been associated with diseases such as glaucoma, edema, epilepsy, obesity, and cancer and is therefore recognized as a drug target. In this review, a brief history of crystallography and its impact on CA research is discussed.

## 1. Introduction

### 1.1. A Brief History of Crystallography

Röntgen discovered a form of radiation in 1895 while analyzing the range of cathode rays in vacuum tubes. He termed this radiation X-rays and determined that the permeability of an object to such radiation directly correlates to its density [1]. Laue hypothesized that radiation of short wavelengths, such as X-rays, would diffract when passed through a crystal if the wavelength is of similar magnitude to the distance between planes of the crystal lattice. Friedrich and Knipping confirmed Laue's hypothesis and successfully demonstrated diffraction from crystals of copper sulfate in 1912 [2]. Based on their findings, Bragg recognized that the treatment of a diffraction pattern as reflections from parallel planes within a crystal lattice could be used to relate the angle of the incident beam to the wavelength and distance between the planes, now known as Bragg's Law [3]. Subsequent experiments led to Bragg's development of the first X-ray spectrometer and determination of the crystal structure of sodium chloride [4, 5].

The growth of protein crystals dates back as early as 1840 with the observation of hemoglobin crystals in blood samples [6]. However, the first crystal of an enzyme, urease, was not achieved until 1926 [7]. X-ray diffraction of a pepsin protein crystal was collected in 1934 following the optimization of conditions to ensure hydration of the crystal during data collection. However, the pepsin structure was not determined for several more decades [8]. The first protein crystal structures determined include myoglobin in 1957 and hemoglobin in 1960 [9, 10].

Nearly a decade after the first observation of X-ray diffraction, interest in obtaining neutron diffraction from single crystals increased. X-ray crystallography requires electrons in the sample to interact with the incoming X-ray beam to generate a diffraction pattern. The scattering factor of an atom is the likelihood of a diffraction event occurring and is dependent on how many electrons are in the atom. Electron rich atoms have a high scattering factor, meaning they are easily distinguishable from the diffraction pattern [11]. Therefore, a resulting limitation of X-ray crystallography is the inability to see hydrogens due to an inherently low scattering factor. However, neutron scattering lengths of Hydrogen and Deuterium are comparable to other atoms. Neutron crystallography can therefore be used to identify the position and accessibility of H atoms, providing insight into side chain protonation states and hydrogen bonding networks that may improve the understanding of catalytic mechanisms [12–14]. Early experiments were performed at the Argonne and Clinton Laboratories, sources used to collect data for the Manhattan Project. Successful neutron diffraction was collected from calcite crystals in 1944 and NaCl crystals in 1945 [15, 16]. The first single crystal neutron structures were determined in 1951 [17].

As interest in protein structure and function continued to grow, the need for a database of crystal structures led to the development of the Protein Data Bank (PDB, rcsb.org) in 1971. The PDB was started with 7 depositions, including the structures of myoglobin and hemoglobin, and has grown to over 125,000 structures to date [18]. This statistic highlights the importance of X-ray and neutron crystallography as techniques for understanding enzyme structure in relation to catalytic mechanism and the use of such knowledge to guide drug design.

### 1.2. Discovery of Carbonic Anhydrases

In the 1920s, two theories were proposed concerning the transport of CO_2_ in the blood. The most common hypothesis was termed the HCO_3_^−^ theory which stated that CO_2_ is transported to the lungs in the form of HCO_3_^−^. The HCO_3_^−^ is then converted to carbonic acid by proteins in the blood, which dehydrates to release CO_2_. However, the rate of spontaneous carbonic acid dehydration was calculated to be significantly lower than the observed physiological rate of CO_2_ respiration [19]. The rate of CO_2_ production was therefore proposed to be increased by a catalyst present in red blood cells [20]. This catalyst was isolated from ox blood in 1932, determined to be distinct from hemoglobin, and given the name carbonic anhydrase (CA) [21]. Thus, it was concluded that CA catalyzes the reversible hydration of CO_2_ to produce HCO_3_^−^ and a proton.

(1) $$\begin{array}{c} CO_{2}+H_{2}O⇌{{HCO}_{3}}^{-}+H^{+} \end{array}$$

Initial experiments identified two distinct forms of CA (CA B and CA C) [22]. CA B was discovered to be ~7-fold more abundant whereas CA C exhibited higher catalytic activity [23]. Fractionation experiments identified a third form, CA A, that was originally thought to be an artifact of purification due to the similarities of amino acid composition between CA A and CA B [24]. The nomenclature of CA A, CA B, and CA C were reassigned as CA III, CA I, and CA II, respectively.

### 1.3. CAs in the Human Genome

CA I, CA II, and CA III were confirmed as unique CA isoforms through sequence comparison and gene mapping [23, 25–28]. The three genes were mapped onto a cluster of genes on chromosome 8 in the order CA II, CA III, and CA I [29]. With the completion of the human genome, a total of 15 CA isoforms have now been identified [30]. These CA isozymes differ by both cellular localization and catalytic efficiency. CA I, CA II, CA III, CA VII, CA VIII, CA X, CA XI, and CA XIII are cytosolic; CA IV, CA IX, CA XII, and CA XIV are membrane-bound; CA Va and CA Vb are mitochondrial; and CA VI is secreted [31].

### 1.4. CA Families

Since the discovery of mammalian CAs in 1932, 7 genetically unique families have been identified: *α*, *β*, *γ*, *δ*, *ζ*, *η*, and *θ* [21, 32–37]. The CA families all catalyze the reversible hydration of CO_2_ and are therefore classified by differences in structural fold. Furthermore, the tetrahedral coordination of a metal ion to three active site residues and a water molecule is conserved between the classes, but the identity of the metal ion and amino acids differs. The *α* CAs are the most well-studied class as these are expressed in mammals, though examples have been identified in bacteria and protozoa as well. The *α* CAs have a catalytic zinc coordinated by three His residues (x, x+2, x+25, where x=94 in human CA II). The activity and/or overexpression of several isoforms in the *α* class have been associated with human diseases and are therefore recognized as therapeutic targets.

*β* CAs were discovered soon after the *α* class in 1939. The *β* CAs were isolated from plant chloroplasts and have since been identified in eubacteria, algae, and archaea. *β* CAs are dimeric in nature and are the only CA class to exhibit allosteric regulation. *β* CAs are also zinc metalloenzymes; however the zinc is coordinated by two Cys and one His in the active site (x, x+56, x+59, where x=68 in Can2) [38]. Because the catalytic activity of *β* CAs has been shown to be essential to the survival of bacteria and this class is structurally unique from the CAs expressed in humans, *β* CAs are targeted in the design of new antibiotics [39].

*γ* CAs have been found in archaea and bacteria. They are catalytically active as trimers with the metal ion coordinated between the monomers by a His residue from each monomer (x, x+36, x+41, where x=81 in Cam) [40]. Previous studies have hypothesized iron as the physiologically relevant metal ion [41]. However, the enzyme is most active when bound to cobalt and maintains activity in coordination with zinc [42].

The *δ* and *ζ* classes of CA are both expressed in marine diatoms. *δ* CAs were first identified in* Thalassiosira weissflogii* in 1997 [43]. The active site structure of *δ* CAs is hypothesized to be similar to that of the *α* class with a catalytic zinc ion coordinated by three His residues (x, x+3, x+112, where x=114 in TweCA) [32, 44]. *ζ* maintains activity with cadmium or zinc bound, though is expected to preferentially bind cadmium in seawater with low concentrations of zinc. The *ζ* CA active site resembles that of *β* CAs coordinating the metal ion to a His and two Cys residues (x, x+52, x+62, where x=263 in CDCA1) [45].

The *η* and *θ* CAs represent the most recently identified families and therefore little information is known about these classes. The *η* CAs are closely related to and previously thought to belong to the *α* class. It was not until 2014 that sequence and phylogenetic analyses identified the *η* CAs as a unique genetic class [33]. *η* CAs are also unique in that the catalytic zinc is hypothesized to be coordinated by two His and one Gln [46]. To date, *η* CA has only been identified in the Plasmodia species of protozoa, the pathogen that causes malaria, and is therefore recognized as a potential target of antiparasitic agents. *θ* CA was isolated from* Phaeodactylum tricornutum *in 2016 and identified as a CA zinc metalloenzyme due to its CO_2_ hydration and esterase activity [34].

### 1.5. CA Crystal Structures

**Figure 1:**
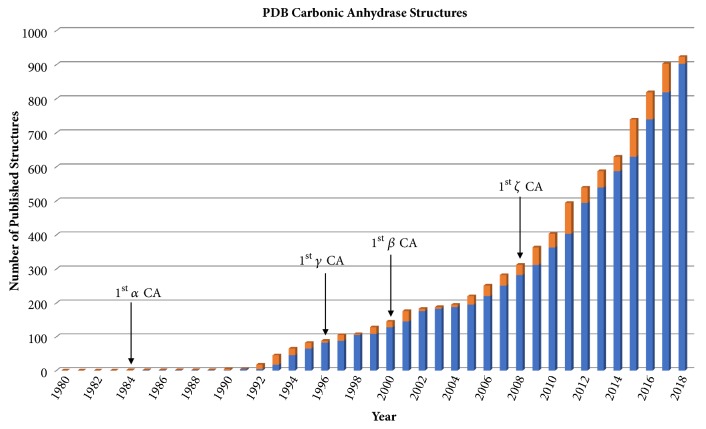
**CA protein database entries** growth of CA PDB entries since 1980. Blue represents all the past years entries whereas orange represents entries submitted that year.

**Figure 2:**
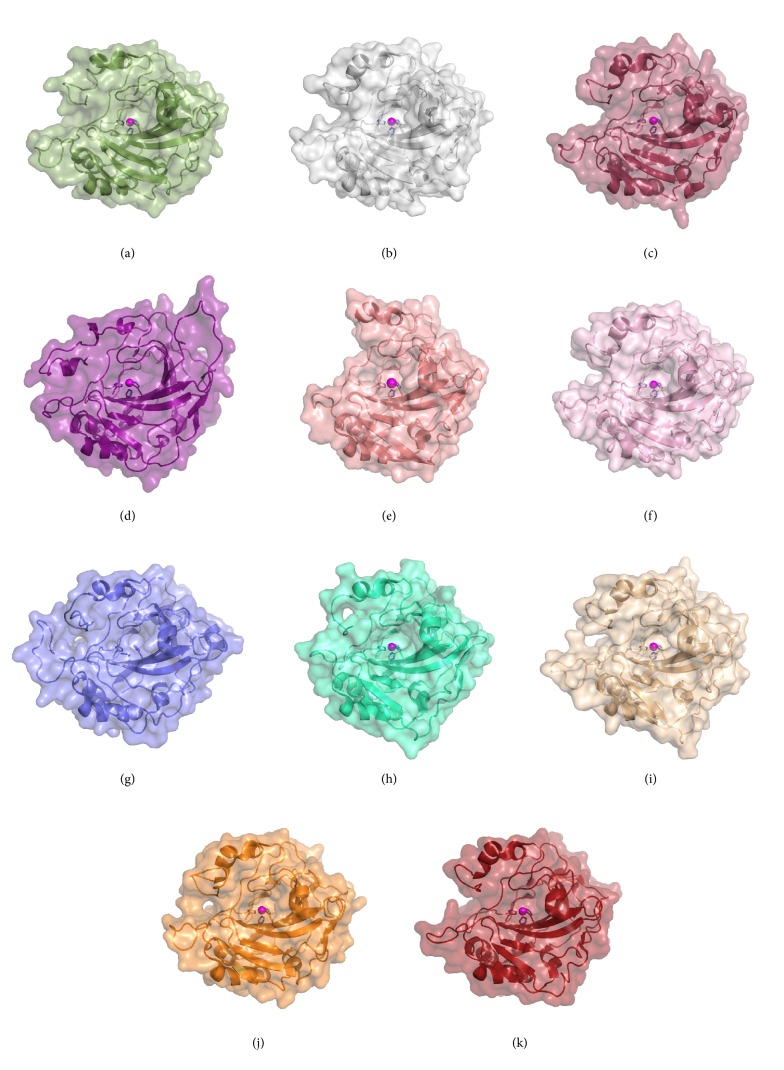
**Human CA isoform structures: (a)** CA I,** (b)** CA II,** (c)** CA III,** (d)** CA IV,** (e)** CAVI,** (f)** CA VII,** (g)** CA VIII,** (h)** CA IX,** (i)** CA XII,** (j)** CA XIII, and** (k)** CA XIV.

To date, crystal structures have only been determined for the *α*, *β*, *γ*, and *ζ* CA families. There are over 900 CA structures deposited in the PDB that have been solved using X-ray or neutron crystallography (Figures 1 and 2). The majority of these structures represent the *α* class, including structures for all the catalytically active human isoforms with the exception of CA V (Figure 2, Table 1) [47–56]. A large number of structures also include *α* CAs in complex with various classes of inhibitors, highlighting the emphasis placed on the *α* CAs as drug targets.

**Figure 3:**
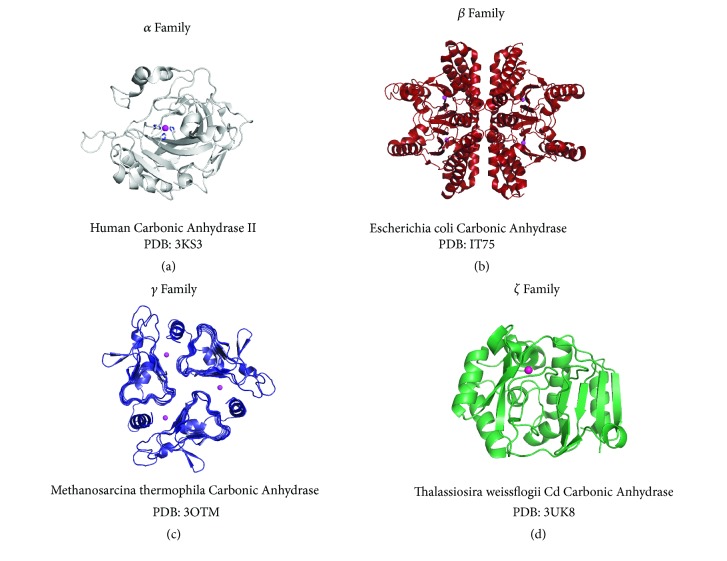
**CA families **structural examples of CAs from** (a)***α* (gray),** (b) ***β* (red),** (c) ***γ* (blue), and** (d)***ζ* (green) families.

The first *α* CA crystal structure of CA II was determined to 2.0 Å resolution by Liljas et al. in 1972 and further refined by Eriksson et al. in 1988 to the space group P2_1_ with cell dimensions* a *= 42.7,* b *= 41.7,* c *= 73.0 Å, and *β* = 104.6°. The overall shape of the molecule is an ellipsoid with dimensions ~40x40x50 Å. The conically shaped active site is ~15 Å deep with the catalytic zinc situated at the base. Seven right-handed *α*-helices are present on the surface of the enzyme, surrounding a 10 stranded *β*-sheet core. The zinc is tetrahedrally coordinated to three His residues and a water molecule (Figure 3(a)). Electron density was observed for six ordered water molecules in the active site that are stabilized via hydrogen bonding with hydrophilic residues [57, 58]. Many CA II structures have since been solved and recently sub-angström resolution was achieved, providing further insight into the CA catalytic mechanism [48]. The CA II active site can be divided into two halves consisting of hydrophobic (I91, V121, F131, V135, L141, V143, L198, P202, L204, V207, and W209) and hydrophilic (N62, H64, N67, Q92, T199, and T200) residues. The hydrophobic region contains the binding site for substrate CO_2_ whereas the hydrophilic residues stabilize the ordered water network that is essential for proton transfer. Dual occupancy of the proton shuttle residue H64 was also observed, which is conserved in catalytic *α* CAs with the exception of CA III that has a Lys [48].

The first crystal structure of a *β* CA isolated from* P. purpureum* red algae was determined in 2000 [35]. Now, nearly 80 structures have been deposited to the PDB representing plant, yeast, bacterial, and archaeal *β* CAs. The *β* CAs form oligomers from dimeric fundamental units and most commonly exist as tetramers with the tetrameric interface perpendicular to that of the individual dimers. All *β* CA monomers exhibit a four or five stranded parallel *β*-sheet core. Upon dimerization, the *β*-sheets of each monomer align to form an extended *β*-sheet. Several *α*-helices surround the core, two of which interact with the other monomer to support dimerization. A hydrophobic cleft along the dimer interface leads to the catalytic zinc and is predicted to bind substrate CO_2_ (Figure 3(b)) [38]. There are two subclasses of *β* CA characterized by the coordination of the zinc ion. In Type I, zinc is coordinated by two Cys residues, one His, and a water molecule/ligand. In Type II the coordination sphere consists of two Cys, one His, and one Asp, which replaces the water. While Type I are active under most pHs, Type II are acatalytic below pH 8.3. This is due to the Asp residue preventing the catalytic hydroxyl from coordinating to the zinc. At basic pHs above 8.3, the Asp residue is removed from the zinc, allowing a water molecule to bind and activate the enzyme [59]. This also results in the formation of an Asp-Arg dyad, similar to the Type I structures, that serves as a hydrogen bond acceptor of the ligand. As the pH reaches 8.3, Type II enzymes undergo conformational changes to mimic the active sites of Type I. A noncatalytic HCO_3_^−^ binding site containing a Trp-Arg-Tyr triad has also been observed in several *β* CAs located approximately 8 Å from the zinc. Occupancy of this site is hypothesized to restrict the Type II class from undergoing conformational change, inactivating the enzyme [60].

Fifteen *γ* CA crystal structures have been deposited in the PDB to date. These structures have all been determined from enzyme purified from* M. thermophila* and* P. horikoshii *of the archaea domain. *γ* CAs are active as homotrimers and each monomer is characterized by a left-handed parallel *β*-helix. The catalytic metal ion binds in the trimer interface and is coordinated to three His residues; H81 and H122 contributed by one monomer and H117 by the other (Figure 3(c)) [41]. This coordination geometry is distorted if zinc or cobalt is bound in place of the physiologically relevant iron. Dual conformations of E84 were identified in the electron density, suggesting a role in proton transfer [36].

Crystals structures of *ζ* CAs are limited to CDCA1. CDCA1 has three tandem repeats (R1-R3), each of which is enzymatically active and mimics the structure of a *β* CA dimer. Each repeat is ellipsoidal in shape with a total of 7 *α*-helices and 9 *β*-strands. The overall structure can be divided into two lobes with a funnel shaped cleft that leads to the active site. The metal ion is tetrahedrally coordinated to three conserved residues (C263, H315, and C325) and a water molecule (Figure 3(d)). A conserved Asp-Arg dyad (D265–R26) contributes to the hydrogen bonding network in the active site and is predicted to contribute to the catalytic mechanism. Interestingly, the apo-CDCA1 structure is stable and has a more open conformation than holo-CDCA1. Rotation of the Cys residues that are no longer coordinating a metal ion contributes to this structural change [37].

## 2. Mechanism

### 2.1. Ping-Pong Mechanism

CAs are some of the most catalytically efficient enzymes known with CA II exhibiting a turnover rate of 1.1* μ*s^−1^ and the fastest, SazCA, a rate of 4.4* μ*s^−1^ [61, 62]. CA II and all subsequent *α* CAs exhibit a classic two-step ping-pong mechanism where the first step is a nucleophilic attack of CO_2,_ generating bicarbonate. The second step is the regeneration of the catalytic zinc-bound hydroxyl [63].

(2) $$\begin{array}{c} ZnOH^{-}+CO_{2}⇌Zn\left(\;OH^{-}\;\right)CO_{2}⇌ZnHC{O_{3}}^{-}⇌\\ ZnH_{2}O+{{HCO}_{3}}^{-}\\ ZnH_{2}O⇌Zn\left(\;OH^{-}\;\right)H^{+}+H64⇌\\ ZnOH^{-}+H64H^{+} \end{array}$$

**Figure 4:**
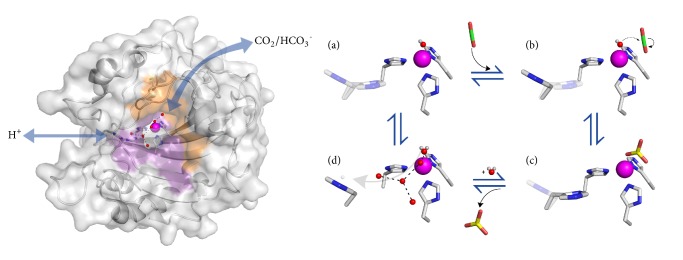
**Active site mechanism. **The surface rendition of CA II with zinc bound to active site residues H94, H96, and H119. The hydrophobic side is depicted in orange while the hydrophilic side is depicted in purple. Blue arrows depict substrate flow, CO_2_, and HCO_3_^−^ through the hydrophobic side and proton transfer through the hydrophilic side. A cartoon depiction of *α* CA ping-pong mechanism.

The full mechanism was first reported in 1988 by Silverman and Lindskog where they described the interworking of the *α* CAs through structure activity relationships and kinetic data (Figure 4) [63]. In the hydration direction, the reaction starts with an open active site, a primed zinc-bound hydroxyl, and an empty hydrophobic binding pocket (Figure 4(a)). CO_2_ diffuses into the active site, binding in a hydrophobic pocket approximately 2.8Å away from the catalytic zinc-bound hydroxyl [64]. As CO_2_ binds, H64 initiates a conformational change from the “out” to the “in” position in preparation for proton transfer [65]. CO_2_ binding triggers a nucleophilic attack from the free pair of electrons on the hydroxyl group onto the partially positive carbon of the CO_2_ (Figure 4(b)) [66]. This forms HCO_3_^−^ anchored to zinc through the same nucleophilic oxygen that performed the attack (Figure 4(c)). HCO_3_^−^ is displaced/released via the binding of a water molecule in its place (Figure 4(d)). This first step is nearly instantaneous due to the efficient nucleophilic attack of the hydroxyl and quick on/off rate of the HCO_3_^−^. It therefore does not significantly contribute to the overall rate of the reaction [63].

The second, rate limiting step is the regeneration of the zinc-bound hydroxyl via proton transfer from the zinc-bound water to bulk solvent [67]. While HCO_3_^−^ is released, H64 completes its conformational change to fully occupy the “in” conformation [67]. An ordered water network in the active site acts as a proton wire, allowing intramolecular proton transfer [68]. This proton wire is made up of six water molecules (ZnH_2_O/OH^−^, DW, W1, W2, W3a, and W3b) that are stabilized through a hydrogen bonding network [68]. Multiple residues located in the hydrophilic region of the active site stabilize these waters, including Y7, N62, N67, T199, and T200 [69]. The zinc-bound water is deprotonated and the proton is moved 8 to 10 Å out of the active site through these clustered waters [69]. These hydrogen bond interactions result in rapid proton shuttling out of the active site and regeneration of the catalytic hydroxyl [68]. Proton transfer is a two-part process. The first being the shuttling of the proton through the six waters to H64. The second is the transfer of the proton between H64 to bulk solvent [70].

### 2.2. Substrate Binding

Due to the highly efficient turnover rate of CA II and the limited solubility of CO_2_ in solution, the binding sites of substrates for CAs remained unknown until hypothesized via molecular dynamics (MD). Using free energy perturbation simulations, Liang and Lipscomb identified three potential CO_2_ binding sites in the hydrophobic region of the active site. One site was identified as the potential catalytic site and two other binding locations likely sites for CO_2_ replenishment [71]. The primary binding pocket was shown to have the highest predicted affinity ~4 Å from the catalytic zinc and interact with residues H119, V121, V143, L198, T199, V207, and W209. Additionally, the determined orientation of CO_2_ in this site supported the mechanism of nucleophilic attack by the zinc-bound hydroxide. To test this proposed binding site, site directed mutagenesis was performed by Fierke et al. [72]. Residues within this hydrophobic pocket were mutated and subsequent kinetic tests were performed. A total of twelve V143 variants of varying size and charge were tested, all of which showed a decrease in catalytic rate. Large aromatic groups such as Phe and Tyr showed the largest impact on activity, resulting in 10^5^ fold decreased rates [72]. Of note, the V143Y mutant decreased catalytic activity below 0.02% of the wild type, confirming that a large, sterically hindering residue in the hydrophobic pocket prevents CO_2_ binding [72]. While convincing, the CO_2_ binding site had yet to be confirmed via X-ray crystallography.

The first CA structures confirming the MD predictions were obtained via cryocooling holo- and apo-CA II under 15 atm of pressurized CO_2_ [73, 74]. Technological advances at synchrotrons allowing data collections under high pressures in conjugation with acidifying of the crystal permitted these structures to be obtained. Under 15 atm of CO_2_, the buffer surrounding the CA crystals becomes acidic, rendering the reaction inactive due to the zinc-bound water preventing nucleophilic attack. As predicted by the MD simulations, the hydrophobic pocket was determined to be the site of CO_2_ binding, approximately 4 Å away from the zinc. This was also true of the apo-CA II. From these structures, it was apparent that T199 forms a weak hydrogen bond with CO_2,_ orienting it for optimal nucleophilic attack [73].

Similarly, the binding site of HCO_3_^−^ in CA II was unknown due to its weak binding affinity. Previously reported work did see success in cobalt substituted structures of CA II, however attempting crystal soaks with wild type CA II at high concentrations of HCO_3_^−^ failed due to the millimolar binding affinity [75]. In 1993, work done by Xue et al. showed CA I exhibited a greater binding affinity for HCO_3_^−^ in relation to CA II. They hypothesized that this difference in affinity was due to the H200 residue, which is a Thr in CA II [76]. Site directed mutagenesis was performed on CA II to make a T200H variant. This variant demonstrated an increase in anion affinities in relation to wild type and also greatly increased HCO_3_^−^ binding affinity [77]. Therefore, the T200H CA II variant was used to determine the first crystal structure of CA II in complex with HCO_3_^−^. The HCO_3_^−^ was observed to bind directly to the zinc, with one of the oxygen atoms occupying the site of the hydroxyl. The other two oxygen atoms bind in the hydrophobic pocket oriented similarly to the previously mentioned CO_2_, one of which displaces an ordered water and forms a hydrogen bond with the NH of T199 [76]. The T199 gatekeeper residue was shown to be important for orienting the substrate as seen previously in the CO_2_ binding studies. T199 is responsible for orienting HCO_3_^−^ for the dehydration reaction [78]. Therefore, it was hypothesized that mutating this residue would have detrimental impacts on CA activity.

To further investigate HCO_3_^−^ binding, West et al. revisited the hydrophobic pocket V143 mutation studies. Combining kinetic assays with O^18^ exchange mass spectrometry, they concluded that a V143A mutation only resulted in a 2-fold decrease in activity, while V143I and V143L resulted in a larger 20-fold decrease [67]. Traditional X-ray crystallography was conducted on all three of these variants; however no changes in the active site were seen. To determine the mutations' influence on the mechanism, the V143I mutant was cryocooled and put under 15 atm of CO_2_ in accordance with previous CO_2_ binding studies [74]. Diffraction data was collected and showed similar CO_2_ binding with the previously reported wild type. However, HCO_3_^−^ was also captured in the active site with an occupancy of 0.35 and CO_2_ with an occupancy of 0.65. This data suggested that the V143I mutation caused a slower conversion of CO_2_ into HCO_3_^−^ with an increased affinity for HCO_3_^−^, allowing it to be captured in the active site. Compared to the previously reported T200H variant and HCO_3_^−^ complex, the V143I showed a 0.4 Å movement in the unbound oxygen atoms and an overall 18° rotation [67]. While similar, this slight movement and rotation were due to increased steric hindrance of the added Ile. This study concluded that the V143I increased an apparent energy barrier of catalysis by ~1.7 kcal/mol owing to the capture of HCO_3_^−^. This is possibly the result of tighter binding of HCO_3_^−^ in the variant as previous CO_2_ capture experiments reported no bound HCO_3_^−^[67, 74].

### 2.3. Proton Transfer

The second step of the *α* CA mechanism, proton transfer, was first hypothesized in 1975 by Steiner et al. Here, they reported that for a realistic mechanism for *α* CAs, the rate limiting step was the regeneration of the zinc-bound hydroxyl [61]. They predicted that there must be an intramolecular transfer of the proton from the zinc-bound water onto a nearby residue, most likely an ionizable His. This concept was tested in 1988 by Silverman and Lindskog during their structural and kinetic testing, hypothesizing that the ordered water network was responsible for the transfer of the proton onto H64 [63]. However, the concept of the proton wire and transfer was just a hypothesis based on the ordered water network found in all CA II crystal structures. There was little to no experimental proof confirming the function of the proton until a series of MD experiments and mutation-based structure activity relationship studies.

The initial MD predictions for the proton wire were performed in 2006 by Fisher et al. Here, they performed a simplified MD simulation, modeling the CA II structure coordinates with 220 locations of water molecules inside a solvated cubic simulation box [70]. The simulation proposed the most likely proton transfer occurring through the 6-membered ordered water network and transferred the proton to H64. The zinc-bound water is stabilized through both T199 and a water termed “deep water” (DW) adjacent to the hydrophobic pocket. This stabilization along with zinc coordination allows a proton to be removed from the water molecule with negligible energy input [70]. The proton is then transferred to W1 and stabilized by the oxygen atom of T200. It is then taken by W2, where it is further stabilized by W3a and W3b. These subsidiary waters do not actually take the proton but instead were predicted to help stabilize the protonated W2. Finally, the simulation estimated that H64 in the “in” confirmation accepts the proton and undergoes a conformational change into the “out” position where the proton is donated to the bulk solvent and out of the active site [70]. While accurate and groundbreaking, this simulation was only modeled using the 6 water cluster, H64, and the zinc-bound solvent, ignoring the rest of the enzyme. This work proved the proton wire was the likely mechanism of proton transfer, but more work was needed to confirm (Figure 4).

Subsequent MD simulations at a higher degree of complexity were performed in an attempt to model the experimentally observed rates of CAs. However, in the early 2000s, many of these experiments produced varying results due to differences in MD methodology [79–81]. In 2009 Maupin et al. used a new technique known as multistate empirical valence bond MD (MS-EVB) to model proton transfer throughout the CA active site that could accurately predict the kinetic rates. Compared to other methods, MS-EVB fully describes the dynamical charge defect delocalization and Grotthuss shuttling of the proton and multiple water molecules [81, 82]. This work also incorporated ionizable moieties that accurately portray zinc-bound water/hydroxide of CA II. Analysis of their MD simulation indicates that the proton travels along a similar path as previously predicted.

(3) Zinc-bound H2O⟶W1⟶W2⟶H64 “in”⟶H64 “out”⟶Bulk Solvent

First, the zinc-bound H_2_O interacts with W1 to form a Zundel cation (H_5_O_2_^+^). The proton is then transferred onto W1, forming a higher energy Eigen cation (H_9_O_4_^+^). The proton is further transferred to W2 as it reverts back to the low energy Zundel cation [81]. However, the highest energy barrier was observed before the proton was transferred to H64, indicating another complex formation with a final Eigen cation generated with W3a. This indicates that, for proton transfer, the limiting rate is Eigen cation formation upon proton transfer to W3a [81].

This MS-EVB MD simulation also accurately predicts H64 proton acceptance and donation. When H64 is in the “out” conformation the free energy barrier for proton transfer is 14.6 ± 0.4 kcal/mol [81]. However, once in the “in” conformation this barrier is lowered to 10.0 ± 0.4 kcal/mol. This decrease in the free energy barrier is due to H64 stabilizing interactions with W2 and the rest of the water network, priming the waters for proton transfer. This energy barrier translates to an estimated catalytic rate of 1.0* μ*s^−1^, matching known catalytic rates of 1.1* μ*s^−1^ [81]. Building on the accuracy of this simulation, the MS-EVB model also accurately predicts the effect of exogenous buffer on the rate of proton transfer. At physiologically relevant conditions, there is essentially an unlimited supply of exogenous buffer for CA to utilize for its proton transfer. At these levels of buffer, the rate limiting step of proton transfer is the transfer of protons through the water cluster and not the H64 release. However, once exogenous buffer is limited, the rate becomes more dependent on H64 proton release, as the limited buffer is less accepting of protons [81, 83]. The MS-EVB simulation accurately predicts this, as the predicted rate of buffer limited conditions drastically decreases to 0.03* μ*s^−1^ compared to the rate of physiological buffer conditions of 1.0* μ*s^−1^.

To confirm the MS-EVB MD simulations, further experiments were conducted to mutate H64 and confirm the importance of proton transfer. H64 was mutated in CA II to Ala and a combination of kinetic, structure, and MD experiments were conducted to determine the effect [84]. Initially, a kinetic test was done on the H64A variant to determine the effect on turnover rate. Using O^18^ mass spectrometry, H64A variant exhibited a 20-50-fold decrease in rate. The effect of having a hydrophobic residue at the point of proton donation resulted in a destabilized water network with W3b being completely absent in the structure. This caused the rate to be dependent on H_2_O deprotonation occurring spontaneously. MS-EVB simulations predicted that other “self-rescuing” pathways were used to shuttle protons, utilizing acidic residues near the active site for proton transfer, forgoing the typical 6 water cluster [84]. 4-Methylimidazole (4MI), a known activator of CAs and potential proton acceptor, was used as a chemical rescue test in kinetic and MD experiments. Again, O^18^ mass spectrometry was used to determine the effects of rescuing H64A variant with 4MI. The 4MI was able to restore functional levels of enzyme activity without the normal H64. The variant was then crystallized in complex with 4MI to determine how its activating effects could rescue proton transfer. Using a combination of NMR and X-ray determined binding sites, the 4MI efficiently restored proton transfer in the MD simulation, further validating the importance of a proton acceptor residue [84].

### 2.4. Neutron Crystallography

While X-ray crystallography is utilized to determine the solvent molecule positions throughout the structure, the methodology is unable to determine their orientation. The clustered water orientations are needed to validate the proton wire as this mechanism largely depends on distinct orientations of the waters, allowing them to span a network of hydrogen bonds. Recent work done by Fisher et al. performed neutron crystallography on CA II at varying pHs to discover the orientations of water in the proton wire [85, 86]. Data collected from crystals at pH 10.0 showed a broken proton wire that lacked a hydrogen bond between W1 and W2. The W2, W3a, and W3b were oriented toward the imidazole ring of H64 occupying the in conformation but did not form hydrogen bonds as previously predicted [85]. However, at pH 7.8, the orientation of W1 changed. At pH 10, W1 served only as a hydrogen bond donor to both DW and T200 whereas at pH 7.8, W1 acts as a hydrogen bond acceptor of T200, forming a subsequent hydrogen bond with W2 [85, 86]. This additional hydrogen bond forces W2 and W3a to reorient in a concerted fashion to complete the proton wire. W2 now behaves as a hydrogen bond donor to H64, indicating that the most likely path for proton transfer is ZnH_2_O/OH^−^-W1-W2-H64 and confirming previous MD simulations and predictions [85, 86].

## 3. Drug Design

### 3.1. *α* CAs as a Drug Target

Multiple *α* CA isoforms are recognized as therapeutic targets in diseases such as glaucoma, edema, epilepsy, obesity, and cancer. Therefore, a significant portion of CA research has focused on the design of CA inhibitors (CAIs). CAI research began in 1940 with the observation that sulfanilamide (*p*-aminobenzene-sulfonamide) inhibited CA activity. Furthermore, this inhibitory effect was shown to relate directly to the sulfonamide group (SO_2_NH_2_) with a loss of inhibition upon its removal [87]. Sulfonamides and their bioisosteres remain the primary scaffold in CAI design and are referred to as the classical CAIs. Sulfonamide-based inhibitors bind to the catalytic zinc in a tetrahedral geometry, displacing the zinc-bound water/hydroxyl. The sulfonamide zinc binding group (ZBG) also forms hydrogen bonds with T199 and E106, two active site residues conserved amongst the *α* CAs and referred to as “gate keepers” [88].

**Figure 5:**
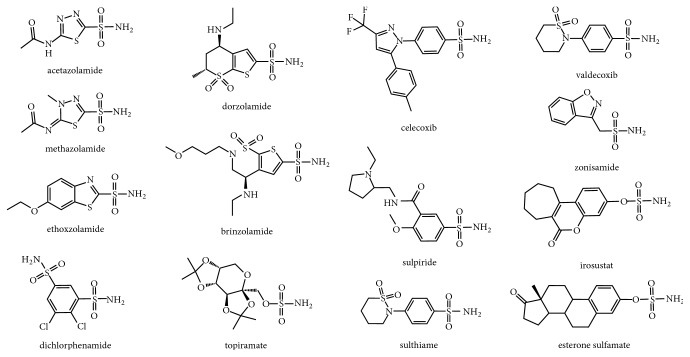
**CA inhibitors** structural formulae of clinically relevant CAIs.

Subsequent studies revealed that heterocyclic sulfonamides exhibited >1,000-fold increase in affinity in comparison to benzene derivatives with a general increase in activity as the acidity of the compound increased [89]. These experiments led to the use of a drug design strategy termed the “ring approach” in which a ring system was added to a sulfonamide ZBG in order to increase affinity for the target CA [90]. Early CAI research led to the development of first generation CAIs that are still available in the clinic, including acetazolamide (Diamox), methazolamide (Neptazane), ethoxzolamide, and dichlorphenamide (Keveyis). However, many of these compounds exhibited solubility issues. Subsequent CAI design therefore employed the “tail method” in which functional groups were added to an aromatic/heterocyclic sulfonamide scaffold in order to modulate the physicochemical properties and solubility of the compound [90]. This technique resulted in the development of second generation CAIs such as dorzolamide (Trusopt) and brinzolamide (Azopt). There are several additional sulfonamide-based CAIs clinically available for the treatment of the aforementioned diseases, including topiramate, celecoxib (Celebrex), sulpiride (Dogmatil), sulthiame, valdecoxib, zonisamide, irosustat (COUMATE), and esterone sulfamate (EMATE) (Figure 5). The role of CA in the onset or progression of each disease and the use of CAIs as a treatment will be discussed.

#### 3.1.1. Glaucoma

Glaucoma is a disease characterized by high intraocular pressure (IOP) and loss of vision. An increase in IOP is most often associated with the retention of aqueous humor, a liquid between the cornea and lens, which is caused by an absence or decrease in humor drainage. A main component of this aqueous humor is sodium bicarbonate, the secretion of which is controlled by CA activity in the uvea of the eye. The inhibition of CA II, CA IV, and CA XII has been shown to decrease the secretion of humor, resulting in decreased IOP. Therefore, CA inhibitors are used in conjunction with adrenergic agonists or antagonists for the treatment of glaucoma [91].

The first CAIs used to treat glaucoma include acetazolamide, methazolamide, ethoxzolamide, and dichlorphenamide. However, these compounds act systemically, inhibiting multiple CA isoforms, and have been shown to cause unwanted side effects such as fatigue, abnormal tingling, and kidney stones [66, 92]. The second generation of compounds, brinzolamide and dorzolamide, exhibit nanomolar inhibition and show increased water solubility to allow for use as a topical agent, resulting in fewer unwanted side effects. However, the drug solution is acidic and can cause redness and irritation. Dorzolamide has also been recorded to exhibit some serious psychological side effects such as depression and dementia [93].

Recent developments in antiglaucoma inhibitors use the tail method with an aromatic sulfonamide scaffold to which amino, hydroxyl, or nitrate ester groups are added to increase solubility. These novel compounds have been tested as topical agents in animals and show good solubility and inhibitory effects, resulting in prolonged decreased IOP. In addition, compounds that contain a NO donating moiety can also aid in vasodilation and humor secretion, supplying more blood to the optic nerve and further decreasing IOP. These so-called “hybrid drugs” were also tested in animals and exhibited a greater reduction in IOP than either brinzolamide or dorzolamide, representing a promising class of lead compounds for antiglaucoma drug development [94].

#### 3.1.2. Edema

Edema is a condition characterized by the retention of fluid, often exhibited as a result of heart failure. Heart failure can decrease blood flow to the kidney, impacting filtration rates and decreasing the secretion of water. CA II, CA IV, CA XII, and CA XIV promote pH homeostasis and bicarbonate resorption in the kidney. CAIs have been proven to act as diuretics by inhibiting the exchange of protons and sodium ions, increasing the concentration of excreted sodium, which is accompanied by movement of water to act as a diluent [95, 96]. Acetazolamide was the first nonmercurial diuretic to be used in the clinic and played a key role in the development of the current drugs [97]. Methazolamide, ethoxzolamide, dichlorphenamide, and thiazide have also been used for the treatment of edema [95, 97]. However, side effects such as fatigue, paresthesia, weakness, or acidosis can lead to renal failure [95]. Patients treated with CAIs often develop a tolerance to the medication, requiring higher doses and increasing the likelihood of undesired side effects. Due to these reasons, CAIs are no longer commonly used as diuretics and have been replaced in the clinic by safer, more efficacious inhibitors.

#### 3.1.3. Epilepsy

Epilepsy is a condition characterized by abnormal brain activity and seizures. It is hypothesized that CA is involved in the secretion of bicarbonate-rich cerebrospinal fluid, similar to CA function in the secretion of aqueous humor in the eye [98]. Although CA II and CA VII are both expressed in neurons, CAI design has mainly focused on the inhibition of CA VII [99]. CA VII is expressed more specifically in the cortex, hippocampus, and thalamus [100]. CA VII activity regulates neuronal pH and maintains bicarbonate gradients in the brain for the management of neuronal channels by GABA receptors (GABARs). GABARs are membrane proteins involved in an array of neuronal pathways and are sensitive to changes in pH, so these receptors are influenced by CA catalytic activity [99, 101]. CA VII is able to quickly replenish bicarbonate, resulting in a net uptake of chloride ions and depolarization, leading to excitation of the GABAR. GABAR excitation and signaling have been shown to promote seizures and CA VII activity has been associated with epilepsy [99]. While the function of CAIs in the brain is not well characterized, the inhibition of CA using acetazolamide has been shown to increase cerebral blood flow and CO_2_ retention [102].

Classic CAIs such as acetazolamide and methazolamide have been clinically used as antiepileptics, but have more recently been replaced by topiramate [98]. Topiramate has a sulfamate moiety that interacts directly with the active site zinc and functions through the enhancement of GABA-mediated inhibition transmissions [103]. However, topiramate has been shown to cause metabolic acidosis with long-term treatments and possibly interfere with bone formation in children, when anticonvulsant therapies are most commonly started [104]. The ring method has been employed to improve antiepileptics by adding valproic acid, a well-known antiepileptic, to aromatic sulfonamide scaffolds such as acetazolamide and topiramate. Compounds with more lipophilic derivatives exhibited better anticonvulsant properties. The valproyl moiety is predicted to interact favorably with the hydrophobic pocket of the active site, making a more efficacious inhibitor. However, the efficacy of CA VII inhibitors greatly decreases if the compound is unable to penetrate the blood brain barrier [98].

#### 3.1.4. Obesity

Significant weight loss has been observed as a side effect of the antiepileptic CAIs topiramate and zonisamide. CA V research is therefore focused on the possibility of a link between CA V inhibition and weight loss. CA V is expressed in the mitochondria as two forms: Vb exhibits wide tissue distribution whereas Va is limited to liver tissue. CA V provides bicarbonate to serve as a substrate for pyruvate carboxylate in gluconeogenesis and oxaloacetate production in lipogenesis. The inhibition of CA V is therefore expected to induce weight loss by depleting a source of bicarbonate and decreasing fatty acid synthesis [105]. Furthermore, the use of a nonspecific CAI, acetazolamide, has been previously shown to decrease the production of fatty acids in human adipose tissue [106]. The majority of the CAIs studied thus far have been substituted benzene sulfonamides with substituents such as amino, hydroxyl, nitro, halogen, and carboxy moieties [107]. The tail method has also been employed to add lipophilic moieties to increase membrane permeability, ensuring the compounds are able to reach mitochondria within the cytoplasm [108]. Kinetic studies have shown that the CA V active site may accommodate bulkier inhibitors and that the location of the additional substituent has more of an effect on the inhibition constant than the chemical identity of the moiety [107, 108].

#### 3.1.5. Cancer

CA IX overexpression has been observed in several cancer types including lung, renal, brain, colon, pancreatic, liver, breast, esophageal, ovarian, and skin cancer. In contrast, CA IX expression is limited to the GI tract in healthy tissue [109]. CA IX expression is regulated by hypoxia inducible factor (HIF-1). Rapidly proliferating tumor cells often develop regions of hypoxia characterized by low oxygen concentrations. In such conditions, the HIF-1*α* subunit is not hydroxylated, preventing recognition by the von Hippel Lindau (VHL) protein and subsequent ubiquitin degradation. HIF-1*α* accumulates and transports into the nucleus where it dimerizes with HIF-1*β* to form an active transcription factor. HIF1 regulates the expression of stress response genes, including CA IX, by binding to the hormone response element [110].

CA IX activity is hypothesized to be critical for the regulation of pH in cancer cells that must thrive in an acidic tumor microenvironment [111–113]. The rapid growth and proliferation of aggressive, metastatic cancers often induce a metabolic switch from oxidative phosphorylation to anaerobic glycolysis, termed the Warburg effect. This metabolic switch increases the amount of lactic acid that is produced and exported by the cell, acidifying the extracellular milieu to pH ~6.5 [114–116]. A decrease in pH impacts cell viability by disrupting biological activities such as ATP production, cell migration, proliferation, and protein synthesis [117]. The overexpression of CA IX enables neoplastic cells to survive such harsh conditions by catalyzing the production of bicarbonate, which can act as a buffer in the surrounding environment or be transported into the cell to maintain near physiological pH [66]. As such, CA IX has been identified as a biomarker and therapeutic target for various cancer types, with a primary focus on breast cancer. Furthermore, the inhibition of CA IX activity via knock down or small molecule inhibition has been shown to decrease tumor volume and improve overall survival in breast cancer mouse models [118].

As CA IX is a membrane-bound isoform with an extracellular catalytic domain, CAI selectivity can be enhanced by designing membrane impermeable compounds to prevent the off-target binding of cytosolic CAs. For example, positively or negatively charged hydrophilic moieties can be added to promote impermeability. However, these properties make it unlikely that such compounds would enter the bloodstream or be developed into a drug to be taken orally. Therefore, inhibitors are designed as prodrugs with hydrophobic moieties that mask the desired, inhibitory substituents until the compound is present in the reductive conditions of a hypoxic environment where it will be hydrolyzed and become an active inhibitor [119].

Artificial sweeteners have recently been identified as potential CAIs for the development of cancer therapeutics. Saccharin and acesulfame potassium were shown to bind within the CA active site and exhibit binding affinities in the micromolar range [120, 121]. Moreover, two sulfonamide-based CAIs, SLC-0111 (4-(4-fluorophenylureido)-benzenesulfonamide) and indisulam (N-(3-chloro-7-indolyl)-1,4-benzenedisulfonamide), have successfully completed phase I clinical trials and are currently entering phase II [122, 123].

### 3.2. Isoform Selective Drug Design

Although several CAIs are currently in use for the treatment of diseases, these clinically available compounds do not exhibit sufficient isoform selectivity and therefore bind to multiple CA isoforms. Off-target binding can lead to sequestration of the drug, requiring higher doses for treatment and subsequently decreasing efficacy. However, the design of isoform specific CAIs is complicated by the structural homology shared by the catalytically active CAs. X-ray and neutron crystallography are therefore utilized to analyze the binding of inhibitors in the CA active site and guide drug design of isoform specific inhibitors.

#### 3.2.1. Structure-Based Drug Design

Structure-guided drug design is a technique that uses high resolution crystal structures of a molecular target to rationalize the design of high affinity, small molecule inhibitors. This process often begins with high throughput screening of different classes of inhibitors to identify lead compounds that inhibit CA activity. The most promising compounds, which exhibit binding affinities in the nano- to micromolar range, are then studied using X-ray crystallography. The crystal structure complex is analyzed to identify interactions between the compound and target molecule that promote selective binding. New derivatives can then be designed to promote such interactions via the addition of functional groups to the lead compound [124].

The tail approach, previously described as a method to improve inhibitor solubility, can also be applied in structure-guided drug design. The mapping and comparison of active site residues between isoforms have identified residues unique to the target isozymes. Elongation or derivatization of a compound tail can promote interactions with these isoform unique residues, improving selectivity [66]. New inhibitors can also be designed using a fragment approach in which small molecules that bind different areas of the active site are chemically linked to synthesize a single compound. This technique has the potential to combine a molecule with high affinity for the active site zinc to a fragment that binds to isoform unique residues at the entrance of the active site [125].

It is important to recognize that the affinity of a CAI is dependent on the free energy of binding (ΔG = ΔH - T ΔS) with both enthalpic and entropic contributions. A ligand loses rotational freedom upon binding, decreasing ΔS. This entropic penalty can be counteracted by enthalpic gains upon the formation of interactions between the ligand and target molecule in addition to increases in entropy as water molecules are displaced from the active site [126]. Both terms of the free energy calculation must therefore be considered during the selection of chemical moieties in the drug design process. For example, the addition of hydrophobic functional groups has been shown to induce entropic penalties that are not balanced by the increase in enthalpy, decreasing the binding affinity for the target [127]. One must also consider how compound derivatization will impact drug delivery and pharmacological properties such as absorption, distribution, metabolism, and excretion. Compounds that are to be delivered orally should follow Lipinski's rule of 5: fewer than 5 Hydrogen bond donors and 10 acceptors, molecular weight < 500 g/mol, and the log P < 5 [128]. However, these are not steadfast rules and many FDA approved drugs do not fulfill each of the five rules [129].

#### 3.2.2. Selective Pocket

**Figure 6:**
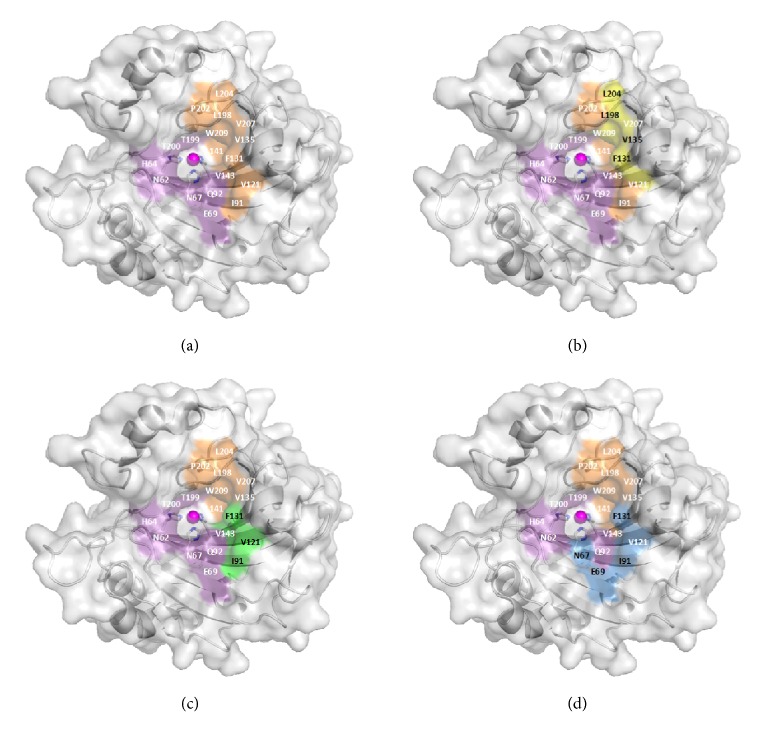
**Pockets in** **α** **CAs. (a)** Hydrophilic and hydrophobic regions of the active site have been colored purple and orange, respectively. The hydrophobic pockets** (b)** 1 and** (c)** 2 have been shaded yellow and green, respectively.** (d)** Isoform unique residues that constitute the selective pocket are shown in blue (Table 2).

The combination of high resolution X-ray and medium resolution neutron crystal structures has led to a thorough characterization of the *α* CA active site as described above. Nearly 500 structures of CA II in complex with CAIs have been deposited into the PDB, allowing the differentiation of compound binding that can be attributed to functionalization or derivatization of lead compounds. For example, shorter inhibitors that contain aromatic functional groups orient into the hydrophobic region of the active site (I91, V121, F131, V135, L141, V143, L198, P202, L204, V207, and W209) and are often involved in parallel stacking interactions with F131. Alternatively, compounds with longer tails or charge were shown to bind preferentially in the hydrophilic region (N62, H64, N67, Q92, T199, and T200) (Figure 6(a)) [130].

The hydrophobic half of the active site was then further divided into two pockets in which the tails of inhibitors orient. Pocket 1 consists of residues L198, F131, V135, and L204 whereas Pocket 2 contains I91, V121, and F131 (Figures 6(b) and 6(c)). The bulky, aromatic side chain of F131 separates the two pockets and often dictates inhibitor binding. The superposition of approximately 30 CAIs identified that the majority of compound tails that bind in the hydrophobic region orient into Pocket 2. However, compound binding affinities were not observed to correlate to pocket preference [131]. Crystallographic studies showed that more flexible linkers, such as ureido fragment (NHCONH), promote tail binding in Pocket 1 and increase affinity [132].

In 2013, a structural comparison of the binding of all nonredundant inhibitors in complex with CA II was performed. Of the 145 compounds, only 14 were observed to orient toward a region between the hydrophobic and hydrophilic areas of the active site. This region was termed the “selective pocket” due to the variability of residues 67, 69, 91, and 131 between isoforms (Figure 6(d)) [133]. Therefore, this pocket can be targeted during the design of CAIs to improve isoform selectivity.

#### 3.2.3. Neutron Crystallography

The use of neutron crystallography in conjunction with X-ray crystallography allows the visualization of both “heavy” (non-H) and “light” (H) atoms. It is therefore possible to determine the protonation state of amino acid side chains and inhibitors using joint refinement. For example, the standard CAI acetazolamide exhibits three possible protonation states in solution. Initial solution state NMR studies hypothesized that sulfonamide-based compounds bind in the deprotonated state and interact with catalytic zinc through the sulfonamide nitrogen [134]. Joint neutron/X-ray studies confirmed that acetazolamide binds in the anionic form with the negatively charged sulfonamido coordinating to the zinc. Neutron crystallography can also provide insight into hydrogen bonding and the displacement of water molecules, which can be used to guide the design of new CAIs [135]. For example, the neutron structure of acetazolamide was compared to methazolamide. The addition of the hydrophobic methyl group prevented the formation of a hydrogen bond and resulted in the displacement of four additional water molecules in comparison to acetazolamide binding. However, the gain in entropy is hypothesized to counteract the loss of hydrogen bonding, justifying the similar binding affinities of the two CAIs [136].

#### 3.2.4. Nonclassical CAIs

The search for isoform selective CAIs has also led to the discovery of nonsulfonamide-based inhibitors that exhibit unique mechanisms of inhibition, including compounds that anchor to the zinc-bound water and CAIs that bind outside the active site, occluding entrance of substrate. These compounds have the additional benefit of preventing adverse effects in individuals with sulfa allergies [88, 137].

Compounds that anchor through the zinc-bound water deselect from zinc binding that is well established for sulfonamide-based inhibitors. Therefore, the binding affinity relies primarily on interactions with residues in the active site. In comparison to compounds that bind directly to the catalytic zinc, inhibitors that anchor through the water extend an additional 2.5–3 Å from the zinc, increasing the probability of forming interactions with isoform unique residues in the selective pocket. Examples of such compounds include phenol- and carboxylic-based inhibitors. Both classes anchor to the zinc-bound solvent through the hydroxyl moiety. Phenol-based compounds exhibit binding affinities within the micromolar range and are hypothesized to inhibit activity via obstruction of the CO_2_ binding site caused by interactions of the phenyl group with hydrophobic residues in the active site [138]. Increasing the length or complexity of phenols, as seen in phenol-based natural products, has been shown to improve inhibitory properties against CAs [139]. Similarly, the alteration of scaffold size and chemical properties in carboxylic acid CAIs has been shown to dictate the orientation of the tail in the active site, promoting isoform selectivity based upon steric hindrance of active site residues. Again, compounds of increasing length that extend toward the selective pocket have been shown to improve the binding affinity over 100-fold [140].

The majority of isoform unique residues encircle the entrance of the active site. Therefore, compounds that inhibit activity by occlusion of the active site are more likely to interact with these unique residues, improving isoform selectivity of the CAIs. Examples of this class of inhibitors include disaccharides and artificial sweeteners, such as sucrose and sucralose. Interestingly, sucralose inhibits CA activity in the micromolar range whereas sucrose binds but does not inhibit CA activity. Based on the crystal structures, sucralose binds along the hydrophobic region and is predicted to prevent entry of CO_2_. In contrast, a small opening into the active site exists when sucrose is bound, allowing movement of CO_2_ [141, 142].

### 3.3. CAIs as Antibiotics

Bacterial resistance is a growing issue worldwide and pathogenic strains such as* S. aureus*,* M. tuberculosis*,* H. pylori*,* B. suis*, and* S. pneumoniae* no longer respond to classic antibiotics [143]. The need to identify new molecular targets essential to the life cycle and virulence of the pathogen has led to a focus on inhibition of bacterial CAs. Because the majority of bacterial CAs belong to the *β* CA class that is not expressed in humans, these enzymes represent promising drug targets. Additionally, CAIs would represent a different mechanism of action compared to current antibiotics and would therefore be less likely to result in resistance [39].

Sulfonamide-based compounds have been shown to inhibit the activity of *β* CAs both in vitro and in vivo in the* H. pylori*,* S. pneumoniae*, and* B. suis* bacterial [144–147]. However, many CAIs that exhibit favorable inhibition profiles in vitro have not been observed in vivo, which is hypothesized to be due to the fact that the CAIs are impermeable to the cell wall [148]. Because sulfonamides also inhibit the *α* CAs expressed in humans, other classes of small molecules have been explored such as phenols, boronic acids, and anions [39]. A promising class includes dithiocarbamate inhibitors that coordinate to the metal ion through the CS_2_^−^ moiety. Aryl, arylalkyl-, heterocyclic, and aliphatic derivatization were shown to increase affinity for *β* CAs isolated from* M. tuberculosis* with some compounds binding in the sub-nanomolar range [149]. Although these compounds exhibit micromolar binding affinities, carboxylic acids have also been under investigation due to the ease of derivatization. Increasing the hydrophobicity of this group of CAIs could potentially improve cell wall permeability [150].

## 4. Conclusion

CAs are an essential class of enzymes in every class of life, from marine diatoms and bacteria to humans. The crystal structure of CA II was amongst the first structures to be determined and was one of the seven structures that contributed to the development of the PDB. Structural characterization has not only led to the classification of the seven CA families but has also improved the understanding of the catalytic mechanism. X-ray and neutron crystallography studies have identified the binding sites of both substrate/product and elucidated proton transfer through observation of an ordered water network and dual conformations of the proton shuttle residue. These techniques have also driven the design of CAIs as antiglaucoma, antiepileptic, antiobesity, and anticancer therapies in addition to antibiotics.

**Figure 7:**
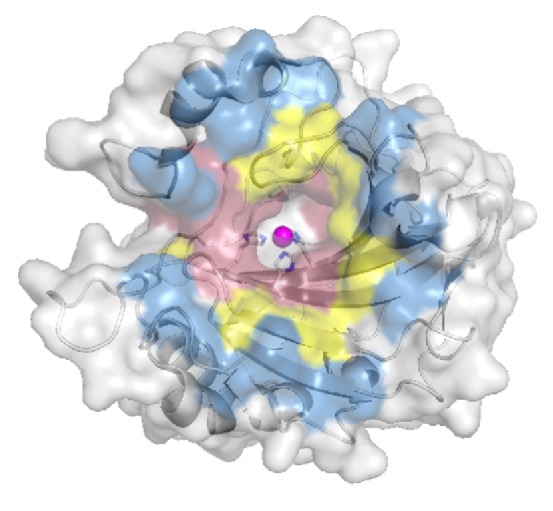
**Selective zones for human** **α** **CAs **The three colored zones represent isoform unique residues in human CAs. Zones are located 5-10 (pink), 10-15 (yellow), and 15-20 (blue) Å from the catalytic zinc (Table 2).

Although structure-guided drug design has led to several classes of high affinity CAIs, the majority of these compounds still do not exhibit sufficient isoform selectivity to prevent off-target binding. Therefore, interactions between an inhibitor tail and the four residues of the selective pocket do not adequately differentiate between the fifteen human *α* CAs. Structural biologists are now mapping residues beyond the active site. CA can be divided into three zones, 5–10 Å, 10–15 Å, and 15–20 Å extending radially from the catalytic zinc (Figure 7, Table 2). With the exception of residue 67, the majority of residues in the 5 – 10 Å zone remain conserved between the isozymes. This observation is reinforced by the lack of selectivity observed for compact CAIs that bind deep within the active site. The 10 – 15 Å zone contains the selective pocket and has therefore been the targeted region for drug design over the past several years. However, the number of isoform unique residues increases further in the 15 – 20 Å zone. In addition to the functionalization of a compound tail, the extension of the linker and/or tail components of CAIs is predicted to further increase selectivity by promoting interactions with the diverse residues outside the active site.

Our current knowledge of the catalytic mechanism is limited to static crystal structures and a complete understanding of the dynamics of the reaction has yet to be achieved. Insight into the dynamics of the proton wire has been recently expanded by the determination of X-ray crystal structures from crystals under high CO_2_ pressure. Varying the time of incubation at room temperature prior to data collection provides a method to visualize CO_2_ release. This study identified an extension of the active site water network termed the entrance conduit waters (EC1, EC2, EC3, EC4, and EC5). Analysis of the alternate conformations of these dynamic water molecules led to the proposal of a restoration of the zinc-bound water following HCO_3_^−^ diffusion [151].

The development of X-ray free electron laser (XFEL) sources now allows the design of serial femtosecond crystallography (SFX) experiments on a time scale capable of capturing intermediate states of a catalytic mechanism [152]. With one of the fastest known biological reactions, CA is an ideal candidate for such SFX experiments to map the movements of substrate/product through the active site. Microcrystals of CA II suitable for XFEL data collection have recently been produced and confirmed to diffract at the Pohang Accelerator Laboratory XFEL [153]. A further benefit is the collection of diffraction data at room temperature before the onset of radiation damage. Data collection at cryogenic temperatures may limit occupancy of alternate conformations relevant to the catalytic mechanism [154]. Alternatively, the collection of multiple data sets at increasing temperatures, known as multitemperature or temperature jump crystallography, allows the sampling of conformational heterogeneity [155].

## Figures and Tables

**Table 1:** Crystal structures of catalytically active human CA isoforms.

	**CA I**	**CA II**	**CA III**	**CA IV**	**CA VI**	**CA VII**	**CA IX**	**CA XII**	**CA XIII**	**CA XIV**
#** Structures in PDB**	24	703	5	10	1	2	6	16	13	2

**PDB ID**	2CAB	3KS3	1Z93	1ZNC	3FE4	3MDZ	5DVX	1JCZ	3D0N	4LU3

**Resolution (Å)**	2.0	0.9	2.1	2.8	1.9	2.3	1.6	1.6	1.6	2.0

**Space group**	P2_1_2_1_2_1_	P2_1_	P6_5_	C2	P2_1_2_1_2_1_	C222_1_	P2_1_2_1_2_1_	C2	P2_1_	P4_1_2_1_2

**Cell dimensions (Å, **°**)**	*a* = 81.5 *b* = 73.6 *c* = 37.1	*a* = 42.3 *b* = 41.4 *c* = 72.3 *β* = 104.4	*a* = 44.7 *b* = 44.7 *c* = 222.6	*a* = 89.5 *b* = 47.7 *c* = 141.0 *β* = 106.3	*a* = 57.7 *b* = 80.0 *c* = 133.4	*a* = 69.7 *b* = 97.7 *c* = 82.4	*a* = 57.9 *b* = 102.7 *c* = 109.0	*a* = 146.7 *b* = 44.6 *c* = 85.2 *β* = 94.1	*a* = 57.8 *b* = 58.2 *c* = 72.1 *β* = 92.4	*a* = 88.6 *b* = 88.6 *c* = 108.9

**Oligomeric state**	Monomer	Monomer	Monomer	Monomer	Monomer	Monomer	Monomer	Dimer	Monomer	Monomer

%** identity to CA II**	61	-	58	34	35	56	34	36	61	37

**Rmsd to CA II (Å)**	0.9	-	0.8	1.3	1.4	0.6	1.4	1.2	0.8	1.2

To date, no crystal structures of CA VA, CAVB, CA X, or CA XI have been deposited in the PDB.

**Table 2:** Active site residues in CA I- CA XIV.

	**CA I**	**CA II**	**CA III**	**CA IV**	**CA VA**	**CA VB**	**CA VI**	**CA VII**	**CA VIII**	**CA IX**	**CA X**	**CA XI**	**CA XII**	**CA XIII**	**CA XIV**
	P00915	P00918	P07451	P22748	P35218	Q9Y2D0	P23280	P43166	P35219	Q16790	Q9NS85	Q866X7	O43570	Q8N1Q1	Q9ULX7
**5-10 Å**															

**5**	W	W	W	W	C	C	W	W	W	W	W	W	W	W	W
**7**	Y	Y	Y	Y	W	Y	Y	Y	Y	Y	Y	Y	Y	Y	Y
**62**	V	N	N	N	T	N	N	N	D	N	T	T	N	S	N
**64**	H	H	K	H	Y	Y	H	H	H	H	R	R	H	H	H
**65**	S	A	T	S	S	L	T	S	T	S	H	H	S	S	T
**67**	H	N	R	M	M	Q	Q	Q	Q	Q	S	S	K	N	Q
**92**	Q	Q	Q	Q	Q	Q	Q	Q	E	Q	Q	Q	Q	Q	Q
**121**	A	V	V	V	V	V	V	V	I	V	I	I	V	V	V
**141**	L	L	I	I	L	L	L	L	I	L	L	L	L	L	L
**143**	V	V	V	V	V	V	V	V	I	V	V	I	V	V	V
**198**	L	L	F	L	L	L	L	L	L	L	M	L	L	L	L
**200**	H	T	T	T	T	T	T	T	I	T	I	T	T	V	T
**207**	V	V	I	V	V	V	V	V	V	V	A	V	V	V	V
**209**	W	W	W	W	W	W	W	W	W	W	W	W	W	W	W
**244**	N	N	N	N	N	N	N	N	N	N	N	N	N	N	N

**10-15 Å**															

**60**	I	L	L	Q	W	W	V	T	T	R	Y	Y	T	S	H
**69**	N	E	V	L	E	E	S	D	I	T	R	L	N	D	S
**91**	F	I	R	K	K	K	Q	K	Y	L	E	S	T	R	A
**131**	L	F	F	V	Y	F	Y	F	I	V	V	L	A	F	L
**135**	A	V	L	Q	V	A	Q	A	V	L	A	T	S	A	A
**201**	P	P	P	P	P	P	P	P	P	P	P	P	P	P	P
**202**	P	P	P	T	P	P	P	P	P	P	P	P	P	P	P
**204**	Y	L	E	D	T	S	T	S	S	A	Y	S	N	L	Y

**15-20 Å**															

**4**	D	H	E	H	S	T	D	G	E	H	W	W	K	S	H
**19**	L	D	L	L	P	V	H	L	V	V	V	V	K	F	S
**20**	Y	F	F	V	V	D	Y	Y	F	S	N	N	Y	F	Y
**21**	P	P	P	P	S	L	P	P	P	P	S	A	P	P	P
**22**	I	I	N	V	V	V	A	I	D	A	A	A	S	I	E
**57**	K	L	K	W	L	L	F	L	C	L	G	G	F	K	L
**58**	E	R	T	T	Y	H	P	S	E	R	T	T	L	I	D
**72**	D	D	D	N	D	D	S	D	S	P	K	P	S	D	S
**123**	W	W	W	K	W	W	Y	W	W	L	Y	F	Y	W	Y
**130**	S	D	T	N	N	N	S	T	S	R	N	N	D	S	S
**132**	A	G	K	K	K	E	D	G	D	D	T	S	S	V	S
**136**	S	Q	K	D	V	L	D	S	G	G	K	R	N	H	E
**170**	K	K	K	P	K	K	P	K	K	E	K	K	K	K	K
**171**	G	G	G	E	D	D	G	G	G	G	N	N	G	G	D
**173**	R	S	E	S	R	L	R	K	S	E	A	A	E	Q	K

## References

[1] Röntgen W. C. (1896). On a new kind of rays. *Science*.

[2] Friedrich W., Knipping P., von Laue M. (1913). Interference Phenomena with rontgen rays. *Annals of Physics*.

[3] Bragg W. L. (1913). The diffraction of short electromagnetic waves by a crystal. *Mathematical Proceedings of the Cambridge Philosophical Society*.

[4] Bragg W. L. (1913). The structure of some crystals as indicated by their diffraction of X-rays. *Proceedings of the Royal Society A Mathematical, Physical and Engineering Sciences*.

[5] Bragg W. H., Bragg W. L. (1913). The reflection of X-rays by crystals. *Proceedings of the Royal Society A Mathematical, Physical and Engineering Sciences*.

[6] Hünefeld F. L. (1840). *Der Chemismus in der thierischen Organisation: physiologisch-chemische Untersuchungen der materiellen Veränderungen oder des Bildungslebens im thierischen Organismus, insbesondere des Blutbildungsprocesses, der Natur der Blutkörperchen und ihrer Kernchen: ein Beitrag zur Physiologie und Heilmittellehre*.

[7] Sumner J. B. (1926). The isolation and crystallization of the enzyme urease preliminary paper. *Journal of Biological Chemistry*.

[8] Bernal J. D., Crowfoot D. (1934). X-ray photographs of crystalline pepsin. *Nature*.

[9] Kendrew J. C., Bodo G., Dintzis H. M., Parrish R. G., Wyckoff H., Phillips D. C. (1958). A three-dimensional model of the myoglobin molecule obtained by x-ray analysis. *Nature*.

[10] Perutz M. F., Rossmann M. G., Cullis A. F., Muirhead H., Will G., North A. C. T. (1960). Structure of Hæmoglobin: a three-dimensional fourier synthesis at 5.5-Å. Resolution, obtained by X-ray analysis. *Nature*.

[11] Groenewegen P. P., Feil D. (1969). Molecular form factors in X-ray crystallography. *Acta Crystallographica Section A: Foundations of Crystallography*.

[12] Shu F., Ramakrishnan V., Schoenborn B. P. (2000). Enhanced visibility of hydrogen atoms by neutron crystallography on fully deuterated myoglobin. *Proceedings of the National Acadamy of Sciences of the United States of America*.

[13] Blakeley M. P., Langan P., Niimura N., Podjarny A. (2008). Neutron crystallography. *Current Opinion in Structural Biology*.

[14] O'Dell W. B., Bodenheimer A. M., Meilleur F. (2016). Neutron protein crystallography: A complementary tool for locating hydrogens in proteins. *Archives of Biochemistry and Biophysics*.

[15] Zinn W. H. (1944). Report for month ending august 26. *Report No.: CP-2081*.

[16] Wollan E. O., Borst L. B. (1945). Physics section III monthly report for the period ending December 31, 1944. *Report No.: M-CP-2222*.

[17] Peterson S. W., Levy H. A. (1951). The use of single‐crystal neutron diffraction data for crystal structure determination. *The Journal of Chemical Physics*.

[18] Berman H. M., Westbrook J., Feng Z. (2000). The protein data bank. *Nucleic Acids Research*.

[19] Henriques O., Biochem Z. (1928). Die bindungsweise des kohlendioxyds im blute. *Biochemische Zeitschrift*.

[20] Van Slyke D., Hawkins J. (1930). Studies of gas and electrolyte equilibria in blood. XVI. the evolution of carbon dioxide from blood and buffer solutions. *Journal of Biological Chemistry*.

[21] Meldrum N. U., Roughton F. J. W. (1933). Carbonic anhydrase. Its preparation and properties. *The Journal of Physiology*.

[22] Rickli E. E., Ghazanfar S. a., Gibbons B. H., Edsall J. T. (1964). Carbonic anhydrases from human erythrocytes preparation and properties of two enzymes. *Journal of Biological Chemistry*.

[23] Henderson L. E., Henriksson D., Nyman P. O. (1976). Primary structure of human carbonic anhydrase. *Journal of Biological Chemistry*.

[24] Nyman P. O. (1961). Purification and properties of carbonic anhydrase from human erythrocytes. *BBA - Biochimica et Biophysica Acta*.

[25] Andersson B., Nyman P. O., Strid L. (1972). Amino acid sequence of human erythrocyte carbonic anhydrase B. *Biochemical and Biophysical Research Communications*.

[26] Lin K. T., Deutsch H. F. (1973). Human carbonic anhydrases. XI. The complete primary structure of carbonic anhydrase B.. *The Journal of Biological Chemistry*.

[27] Holmes R. S. (1976). Mammalian carbonic anhydrase isozymes : Evidence for A third locus. *Journal of Experimental Zoology*.

[28] HOLMES R. S. (1977). Purification, molecular properties and ontogeny of carbonic anhydrase isozymes: evidence for A, B and C isozymes in avian and mammalian tissues. *European Journal of Biochemistry*.

[29] Lowe N., Edwards Y. H., Edwards M., Butterworth P. H. W. (1991). Physical mapping of the human carbonic anhydrase gene cluster on chromosome 8. *Genomics*.

[30] Lander E. S., Linton L. M., Birren B. (2001). Initial sequencing and analysis of the human genome. *Nature*.

[31] Frost S. C. (2014). Physiological functions of the alpha class of carbonic anhydrases. *Subcellular Biochemistry*.

[32] Del Prete S., Vullo D., Scozzafava A., Capasso C., Supuran C. T. (2014). Cloning, characterization and anion inhibition study of the *δ*-class carbonic anhydrase (TweCA) from the marine diatom Thalassiosira weissflogii. *Bioorganic & Medicinal Chemistry*.

[33] Del Prete S., Vullo D., Fisher G. M. (2014). Discovery of a new family of carbonic anhydrases in the malaria pathogen Plasmodium falciparum - The *η*-carbonic anhydrases. *Bioorganic & Medicinal Chemistry Letters*.

[34] Kikutani S., Nakajima K., Nagasato C., Tsuji Y., Miyatake A., Matsuda Y. (2016). Thylakoid luminal Θ-carbonic anhydrase critical for growth and photosynthesis in the marine diatom Phaeodactylum tricornutum. *Proceedings of the National Acadamy of Sciences of the United States of America*.

[35] Mitsuhashi S., Mizushima T., Yamashita E. (2000). X-ray structure of *β*-carbonic anhydrase from the red alga, Porphyridium purpureum, reveals a novel catalytic site for CO2 hydration. *The Journal of Biological Chemistry*.

[36] Iverson T. M., Alber B. E., Kisker C., Ferry J. G., Rees D. C. (2000). A closer look at the active site of *γ*-class carbonic anhydrases: High-resolution crystallographic studies of the carbonic anhydrase from methanosarcina thermophila. *Biochemistry*.

[37] Xu Y., Feng L., Jeffrey P. D., Shi Y., Morel F. M. M. (2008). Structure and metal exchange in the cadmium carbonic anhydrase of marine diatoms. *Nature*.

[38] Rowlett R. S. (2010). Structure and catalytic mechanism of the *β*-carbonic anhydrases. *Biochimica et Biophysica Acta—Proteins and Proteomics*.

[39] Supuran C. T. (2011). Bacterial carbonic anhydrases as drug targets: toward novel antibiotics?. *Frontiers in Pharmacology*.

[40] Zimmerman S. A., Tomb J.-F., Ferry J. G. (2010). Characterization of CamH from Methanosarcina thermophila, founding member of a subclass of the *γ* class of carbonic anhydrases. *Journal of Bacteriology*.

[41] Ferry J. G. (2010). The *γ* class of carbonic anhydrases. *Biochimica et Biophysica Acta (BBA) - Proteins and Proteomics*.

[42] Alber B. E., Colangelo C. M., Dong J. (1999). Kinetic and spectroscopic characterization of the gamma-carbonic anhydrase from the methanoarchaeon Methanosarcina thermophila. *Biochemistry*.

[43] Roberts S. B., Lane T. W., Morel F. M. M. (1997). Carbonic anhydrase in the marine diatom Thalassiosira weissflogii (Bacillariophyceae). *Journal of Phycology*.

[44] Beauchemin M., Morse D., Supuran C. T., Simone G. D. (2015). Chapter 19 - *δ*-carbonic anhydrases: structure, distribution, and potential roles. *Carbonic Anhydrases as Biocatalysts*.

[45] Amata O., Marino T., Russo N., Toscano M. (2011). Catalytic activity of a *ζ*-class zinc and cadmium containing carbonic anhydrase. Compared work mechanisms. *Physical Chemistry Chemical Physics*.

[46] De Simone G., Di Fiore A., Capasso C., Supuran C. T. (2015). The zinc coordination pattern in the *η*-carbonic anhydrase from Plasmodium falciparum is different from all other carbonic anhydrase genetic families. *Bioorganic & Medicinal Chemistry Letters*.

[47] Kannan K. K., Ramanadham M., Jones T. A. (1984). Structure, refinement, and function of carbonic anhydrase isozymes: Refinement of human carbonic anhydrase I. *Annals of the New York Academy of Sciences*.

[48] Avvaru B. S., Kim C. U., Sippel K. H. (2010). A short, strong hydrogen bond in the active site of human carbonic anhydrase II. *Biochemistry*.

[49] Duda D. M., Tu C., Fisher S. Z. (2005). Human carbonic anhydrase III: structural and kinetic study of catalysis and proton transfer. *Biochemistry*.

[50] Stams T., Nair S. K., Okuyama T., Waheed A., Sly W. S., Christianson D. W. (1996). Crystal structure of the secretory form of membrane-associated human carbonic anhydrase IV at 2.8-A resolution. *Proceedings of the National Acadamy of Sciences of the United States of America*.

[51] Pilka E. S., Kochan G., Oppermann U., Yue W. W. (2012). Crystal structure of the secretory isozyme of mammalian carbonic anhydrases CA VI: implications for biological assembly and inhibitor development. *Biochemical and Biophysical Research Communications*.

[52] Picaud S. S., Muniz J. R. C., Kramm A. (2009). Crystal structure of human carbonic anhydrase-related protein VIII reveals the basis for catalytic silencing. *Proteins: Structure, Function, and Bioinformatics*.

[53] Mahon B. P., Bhatt A., Socorro L. (2016). The structure of carbonic anhydrase IX is adapted for low-pH catalysis. *Biochemistry*.

[54] Whittington D. A., Waheed A., Ulmasov B. (2001). Crystal structure of the dimeric extracellular domain of human carbonic anhydrase XII, a bitopic membrane protein overexpressed in certain cancer tumor cells. *Proceedings of the National Acadamy of Sciences of the United States of America*.

[55] Di Fiore A., Monti S. M., Hilvo M. (2009). Crystal structure of human carbonic anhydrase XIII and its complex with the inhibitor acetazolamide. *Proteins: Structure, Function, and Bioinformatics*.

[56] Alterio V., Pan P., Parkkila S. (2014). The structural comparison between membrane-associated human carbonic anhydrases provides insights into drug design of selective inhibitors. *Biopolymers*.

[57] Liljas A., Kannan K. K., Bergstén P.-C. (1972). Crystal structure of human carbonic anhydrase C. *Nature New Biology*.

[58] Eriksson A. E., Jones T. A., Liljas A. (1988). Refined structure of human carbonic anhydrase II at 2.0 Å resolution. *Proteins: Structure, Function, and Genetics*.

[59] Ferraroni M., Del Prete S., Vullo D., Capasso C., Supuran C. T. (2015). Crystal structure and kinetic studies of a tetrameric type II *β*-carbonic anhydrase from the pathogenic bacterium Vibrio cholerae. *Acta Crystallographica Section D: Biological Crystallography*.

[60] Cronk J. D., Rowlett R. S., Zhang K. Y. J. (2006). Identification of a novel noncatalytic bicarbonate binding site in eubacterial *β*-carbonic anhydrase. *Biochemistry*.

[61] Steiner H., Jonsson B., Lindskog S. (1975). The catalytic mechanism of carbonic anhydrase. hydrogen-isotope effects on the kinetic parameters of the human C isoenzyme. *European Journal of Biochemistry*.

[62] Vullo D., De Luca V., Scozzafava A. (2012). Anion inhibition studies of the fastest carbonic anhydrase (CA) known, the extremo-CA from the bacterium Sulfurihydrogenibium azorense. *Bioorganic & Medicinal Chemistry Letters*.

[63] Silverman D. N., Lindskog S. (1988). The catalytic mechanism of carbonic anhydrase: implications of a rate-limiting protolysis of water. *Accounts of Chemical Research*.

[64] Merz K. M. (1991). Carbon dioxide binding to human carbonic anhydrase II. *Journal of the American Chemical Society*.

[65] Maupin C. M., Voth G. A. (2007). Preferred orientations of His64 in human carbonic anhydrase II. *Biochemistry*.

[66] Frost S. C., McKenna R. (2013). Carbonic anhydrase: mechanism, regulation, links to disease, and industrial applications. *Springer Science & Business Media*.

[67] West D., Kim C. U., Tu C. (2012). Structural and kinetic effects on changes in the CO2 binding pocket of human carbonic anhydrase II. *Biochemistry*.

[68] Boone C. D., Pinard M., McKenna R., Silverman D. (2014). Catalytic mechanism of *α*-class carbonic anhydrases: CO2 hydration and proton transfer. *Carbonic Anhydrase: Mechanism, Regulation, Links to Disease, and Industrial Applications*.

[69] Fisher Z., Hernandez Prada J. A., Tu C. (2005). Structural and kinetic characterization of active-site histidine as a proton shuttle in catalysis by human carbonic anhydrase II. *Biochemistry*.

[70] Fisher S. Z., Maupin C. M., Budayova-Spano M. (2007). Atomic crystal and molecular dynamics simulation structures of human carbonic anhydrase II: insights into the proton transfer mechanism. *Biochemistry*.

[71] Liang J. Y., Lipscomb W. N. (1990). Binding of substrate CO2 to the active site of human carbonic anhydrase II: a molecular dynamics study.. *Proceedings of the National Acadamy of Sciences of the United States of America*.

[72] Fierke C. A., Calderone T. L., Krebs J. F. (1991). Functional consequences of engineering the hydrophobic pocket of carbonic anhydrase II. *Biochemistry*.

[73] Domsic J. F., Avvaru B. S., Chae U. K. (2008). Entrapment of carbon dioxide in the active site of carbonic anhydrase II. *The Journal of Biological Chemistry*.

[74] Domsic J. F., McKenna R. (2010). Sequestration of carbon dioxide by the hydrophobic pocket of the carbonic anhydrases. *Biochimica et Biophysica Acta (BBA) - Proteins and Proteomics*.

[75] Håkansson K., Wehnert A. (1992). Structure of cobalt carbonic anhydrase complexed with bicarbonate. *Journal of Molecular Biology*.

[76] Xue Y., Vidgren J., Svensson L. A., Liljas A., Jonsson B., Lindskog S. (1993). Crystallographic analysis of Thr‐200 → His human carbonic anhydrase II and its complex with the substrate, HCO 3−. *Proteins: Structure, Function, and Bioinformatics*.

[77] Behravan G., Jonsson B., Lindskog S. (1990). Fine tuning of the catalytic properties of carbonic anhydrase. Studies of a Thr200 His variant of human isoenzyme II. *European Journal of Biochemistry*.

[78] Liljas A., Håkansson K., Jonsson B. H., Xue Y. (1994). Inhibition and catalysis of carbonic anhydrase: Recent crystallographic analyses. *European Journal of Biochemistry*.

[79] Riccardi D., König P., Prat-Resina X. (2006). "Proton holes" in long-range proton transfer reactions in solution and enzymes: A theoretical analysis. *Journal of the American Chemical Society*.

[80] Riccardi D., König P., Guo H., Cui Q. (2008). Proton transfer in carbonic anhydrase is controlled by electrostatics rather than the orientation of the acceptor. *Biochemistry*.

[81] Maupin C. M., McKenna R., Silverman D. N., Voth G. A. (2009). Elucidation of the proton transport mechanism in human carbonic anhydrase II. *Journal of the American Chemical Society*.

[82] Maupin C. M., Zheng J., Tu C., McKenna R., Silverman D. N., Voth G. A. (2009). Effect of active-site mutation at Asn67 on the proton transfer mechanism of human carbonic anhydrase II. *Biochemistry*.

[83] Tu C., Silverman D. N., Forsman C., Jonsson B.-H., Lindskog S. (1989). Role of histidine 64 in the catalytic mechanism of human carbonic anhydrase II studied with a site-specific mutant. *Biochemistry*.

[84] Maupin C. M., Castillo N., Taraphder S. (2011). Chemical rescue of enzymes: Proton transfer in mutants of human carbonic anhydrase II. *Journal of the American Chemical Society*.

[85] Fisher S. Z., Kovalevsky A. Y., Domsic J. F. (2010). Neutron structure of human carbonic anhydrase II: Implications for proton transfer. *Biochemistry*.

[86] Fisher Z., Kovalevsky A. Y., Mustyakimov M., Silverman D. N., McKenna R., Langan P. (2011). Neutron structure of human carbonic anhydrase II: A hydrogen-bonded water network "switch" is observed between pH 7.8 and 10.0. *Biochemistry*.

[87] Keilin D., Mann T. (1940). Carbonic anhydrase. Purification and nature of the enzyme. *Biochemical Journal*.

[88] Supuran C. T. (2015). How many carbonic anhydrase inhibition mechanisms exist?. *Journal of Enzyme Inhibition and Medicinal Chemistry*.

[89] Miller W. H., Dessert A. M., Roblin R. O. (1950). Heterocyclic Sulfonamides as Carbonic Anhydrase Inhibitors. *Journal of the American Chemical Society*.

[90] Bozdag M., Ferraroni M., Nuti E. (2014). Combining the tail and the ring approaches for obtaining potent and isoform-selective carbonic anhydrase inhibitors: Solution and X-ray crystallographic studies. *Bioorganic & Medicinal Chemistry*.

[91] Becker B. (1955). The mechanism of the fall in intraocular pressure induced by the CARBONIC anhydrase inhibitor, diamox∗. *American Journal of Ophthalmology*.

[92] Maren T. H. (1967). Carbonic anhydrase: chemistry, physiology, and inhibition. *Physiological Reviews*.

[93] Sugrue M. F. (2000). Pharmacological and ocular hypotensive properties of topical carbonic anhydrase inhibitors. *Progress in Retinal and Eye Research*.

[94] Fabrizi F., Mincione F., Somma T. (2012). A new approach to antiglaucoma drugs: carbonic anhydrase inhibitors with or without NO donating moieties. Mechanism of action and preliminary pharmacology. *Journal of Enzyme Inhibition and Medicinal Chemistry*.

[95] Moyer J. H. (1959). Diuretics. *AJN, American Journal of Nursing*.

[96] Kemp G., Kemp D. (1978). Diuretics. *American Journal of Nursing*.

[97] Supuran C. T. (2008). Carbonic anhydrases: novel therapeutic applications for inhibitors and activators. *Nature Reviews Drug Discovery*.

[98] Masereel B., Rolin S., Abbate F., Scozzafava A., Supuran C. T. (2002). Carbonic anhydrase inhibitors: Anticonvulsant sulfonamides incorporating valproyl and other lipophilic moieties. *Journal of Medicinal Chemistry*.

[99] Ruusuvuori E., Huebner A. K., Kirilkin I. (2013). Neuronal carbonic anhydrase VII provides GABAergic excitatory drive to exacerbate febrile seizures. *EMBO Journal*.

[100] De Luca L., Ferro S., Damiano F. M. (2014). Structure-based screening for the discovery of new carbonic anhydrase VII inhibitors. *European Journal of Medicinal Chemistry*.

[101] Thiry A., Masereel B., Dogné J.-M., Supuran C. T., Wouters J., Michaux C. (2007). Exploration of the binding mode of indanesulfonamides as selective inhibitors of human carbonic anhydrase type VII by targeting Lys91. *ChemMedChem*.

[102] Laux B. E., Raichle M. E. (1978). The effect of acetazolamide on cerebral blood flow and oxygen utilization in the rhesus monkey. *The Journal of Clinical Investigation*.

[103] Casini A., Antel J., Abbate F. (2003). Carbonic anhydrase inhibitors: SAR and X-ray crystallographic study for the interaction of sugar sulfamates/sulfamides with isozymes I, II and IV. *Bioorganic & Medicinal Chemistry Letters*.

[104] Jovanović M., Sokić D., Grabnar I. (2014). Effect of long-term topiramate therapy on serum bicarbonate and potassium levels in adult epileptic patients. *Annals of Pharmacotherapy*.

[105] de Simone G., Supuran C. T. (2007). Antiobesity carbonic anhydrase inhibitors. *Current Topics in Medicinal Chemistry*.

[106] Bray G. A. (1972). Lipogenesis in human adipose tissue: some effects of nibbling and gorging.. *The Journal of Clinical Investigation*.

[107] Innocenti A., Antel J., Wurl M., Scozzafava A., Supuran C. T. (2004). Carbonic anhydrase inhibitors: Inhibition of human cytosolic isozyme II and mitochondrial isozyme V with a series of benzene sulfonamide derivatives. *Bioorganic & Medicinal Chemistry Letters*.

[108] Poulsen S.-A., Wilkinson B. L., Innocenti A., Vullo D., Supuran C. T. (2008). Inhibition of human mitochondrial carbonic anhydrases VA and VB with para-(4-phenyltriazole-1-yl)-benzenesulfonamide derivatives. *Bioorganic & Medicinal Chemistry Letters*.

[109] Mahon B., Pinard M., McKenna R. (2015). Targeting carbonic anhydrase IX activity and expression. *Molecules*.

[110] Supuran C. T. (2017). Carbonic anhydrase inhibition and the management of hypoxic tumors. *Metabolites*.

[111] Sedlakova O., Svastova E., Takacova M., Kopacek J., Pastorek J., Pastorekova S. (2014). Carbonic anhydrase IX, a hypoxia-induced catalytic component of the pH regulating machinery in tumors. *Frontiers in Physiology*.

[112] McDonald P. C., Winum J., Supuran C. T., Dedhar S. (2012). Recent developments in targeting carbonic anhydrase IX for cancer therapeutics. *Oncotarget*.

[113] Li Y., Tu C., Wang H., Silverman D. N., Frost S. C. (2011). Catalysis and pH control by membrane-associated carbonic anhydrase IX in MDA-MB-231 breast cancer cells. *The Journal of Biological Chemistry*.

[114] Warburg O., Wind F., Negelein E. (1927). The metabolism of tumors in the body. *The Journal of General Physiology*.

[115] Chen X., Qian Y., Wu S. (2015). The warburg effect: evolving interpretations of an established concept. *Free Radical Biology & Medicine*.

[116] Gonzalez C. D., Alvarez S., Ropolo A., Rosenzvit C., Bagnes M. F. G., Vaccaro M. I. (2014). Autophagy, warburg, and warburg reverse effects in human cancer. *BioMed Research International*.

[117] Chiche J., Ilc K., Laferrière J. (2009). Hypoxia-inducible carbonic anhydrase IX and XII promote tumor cell growth by counteracting acidosis through the regulation of the intracellular pH. *Cancer Research*.

[118] Lou Y., McDonald P. C., Oloumi A., Chia S., Ostlund C., Ahmadi A. (2011). Targeting tumor hypoxia: Suppression of breast tumor growth and metastasis by novel carbonic anhydrase IX inhibitors (Cancer Research (2011) 71, (3364-3376)). *Cancer Research*.

[119] Carroux C. J., Rankin G. M., Moeker J. (2013). A prodrug approach toward cancer-related carbonic anhydrase inhibition. *Journal of Medicinal Chemistry*.

[120] Murray A. B., Lomelino C. L., Supuran C. T., McKenna R. (2018). "seriously Sweet": acesulfame K exhibits selective inhibition using alternative binding modes in carbonic anhydrase isoforms. *Journal of Medicinal Chemistry*.

[121] Mahon B. P., Hendon A. M., Driscoll J. M. (2015). Saccharin: A lead compound for structure-based drug design of carbonic anhydrase IX inhibitors. *Bioorganic & Medicinal Chemistry*.

[122] Carta F., Vullo D., Osman S. M., AlOthman Z., Supuran C. T. (2017). Synthesis and carbonic anhydrase inhibition of a series of SLC-0111 analogs. *Bioorganic & Medicinal Chemistry*.

[123] Abbate F., Casini A., Owa T., Scozzafava A., Supuran C. T. (2004). Carbonic anhydrase inhibitors: E7070, a sulfonamide anticancer agent, potently inhibits cytosolic isozymes I and II, and transmembrane, tumor-associated isozyme IX. *Bioorganic & Medicinal Chemistry Letters*.

[124] Anderson A. C. (2003). The process of structure-based drug design. *Chemistry & Biology*.

[125] Bienstock R. J. (2011). Overview: fragment-based drug design. *Library design, search methods, and applications of fragment-based drug design*.

[126] Lounnas V., Ritschel T., Kelder J., McGuire R., Bywater R. P., Foloppe N. (2013). Current progress in structure-based rational drug design marks a new mindset in drug discovery. *Computational and Structural Biotechnology Journal*.

[127] Butler J. A. V. (1937). The energy and entropy of hydration of organic compounds. *Transactions of the Faraday Society*.

[128] Lipinski C. A., Lombardo F., Dominy B. W., Feeney P. J. (1997). Experimental and computational approaches to estimate solubility and permeability in drug discovery and development settings. *Advanced Drug Delivery Reviews*.

[129] Zhang M., Wilkinson B. (2007). Drug discovery beyond the ‘rule-of-five’. *Current Opinion in Biotechnology*.

[130] Menchise V., De Simone G., Alterio V. (2005). Carbonic anhydrase inhibitors: Stacking with Phe131 determines active site binding region of inhibitors as exemplified by the X-ray crystal structure of a membrane-impermeant antitumor sulfonamide complexed with isozyme II. *Journal of Medicinal Chemistry*.

[131] Avvaru B. S., Wagner J. M., Maresca A. (2010). Carbonic anhydrase inhibitors. the X-ray crystal structure of human isoform II in adduct with an adamantyl analogue of acetazolamide resides in a less utilized binding pocket than most hydrophobic inhibitors. *Bioorganic & Medicinal Chemistry Letters*.

[132] Pacchiano F., Aggarwal M., Avvaru B. S. (2010). Selective hydrophobic pocket binding observed within the carbonic anhydrase II active site accommodate different 4-substituted-ureido-benzenesulfonamides and correlate to inhibitor potency. *Chemical Communications*.

[133] Aggarwal M., Kondeti B., McKenna R. (2013). Insights towards sulfonamide drug specificity in *α*-carbonic anhydrases. *Bioorganic & Medicinal Chemistry*.

[134] Kanamori K., Roberts J. D. (1983). Nitrogen-15 nuclear magnetic resonance study of benzenesulfonamide and cyanate binding to carbonic anhydrase. *Biochemistry*.

[135] Fisher S. Z., Aggarwal M., Kovalevsky A. Y., Silverman D. N., McKenna R. (2012). Neutron diffraction of acetazolamide-bound human carbonic anhydrase II reveals atomic details of drug binding. *Journal of the American Chemical Society*.

[136] Aggarwal M., Kovalevsky A. Y., Velazquez H., Fisher S. Z., Smith J. C., McKenna R. (2016). Neutron structure of human carbonic anhydrase II in complex with methazolamide: Mapping the solvent and hydrogen-bonding patterns of an effective clinical drug. *IUCrJ*.

[137] Lomelino C., Supuran C., McKenna R. (2016). Non-classical inhibition of carbonic anhydrase. *International Journal of Molecular Sciences*.

[138] Nair S. K., Ludwig P. A., Christianson D. W. (1994). Two-site binding of phenol in the active site of human carbonic anhydrase II: structural implications for substrate association. *Journal of the American Chemical Society*.

[139] Innocenti A., Sarikaya S. B. Ö., Gülçin I., Supuran C. T. (2010). Carbonic anhydrase inhibitors: inhibition of mammalian isoforms I-XIV with a series of natural product polyphenols and phenolic acids. *Bioorganic & Medicinal Chemistry*.

[140] Sechi M., Innocenti A., Pala N. (2012). Inhibition of *α*-class cytosolic human carbonic anhydrases I, II, IX and XII, and *β*-class fungal enzymes by carboxylic acids and their derivatives: New isoform-I selective nanomolar inhibitors. *Bioorganic & Medicinal Chemistry Letters*.

[141] Pinard M. A., Aggarwal M., Mahon B. P., Tu C., McKenna R. (2015). A sucrose-binding site provides a lead towards an isoform-specific inhibitor of the cancer-associated enzyme carbonic anhydrase IX. *Acta Crystallographica Section F: Structural Biology Communications*.

[142] Lomelino C. L., Murray A. B., Supuran C. T., McKenna R. (2018). Sweet binders: carbonic anhydrase IX in complex with sucralose. *ACS Medicinal Chemistry Letters*.

[143] Furtado G. H., Nicolau D. P. (2010). Overview perspective of bacterial resistance. *Expert Opinion on Therapeutic Patents*.

[144] Nishimori I., Minakuchi T., Kohsaki T. (2007). Carbonic anhydrase inhibitors: The *β*-carbonic anhydrase from Helicobacter pylori is a new target for sulfonamide and sulfamate inhibitors. *Bioorganic & Medicinal Chemistry Letters*.

[145] Nishimori I., Onishi S., Takeuchi H., Supuran C. T. (2008). The alpha and beta classes carbonic anhydrases from helicobacter pylori as novel drug targets. *Current Pharmaceutical Design*.

[146] Burghout P., Cron L. E., Gradstedt H. (2010). Carbonic anhydrase is essential for Streptococcus pneumoniae growth in environmental ambient air. *Journal of Bacteriology*.

[147] Winum J.-Y., Köhler S., Supuran C. T. (2010). Brucella carbonic anhydrases: New targets for designing anti-infective agents. *Current Pharmaceutical Design*.

[148] Nishimori I., Minakuchi T., Maresca A., Carta F., Scozzafava A., Supuran C. T. (2010). The *β*-carbonic anhydrases from Mycobacterium tuberculosis as drug targets. *Current Pharmaceutical Design*.

[149] Maresca A., Carta F., Vullo D., Supuran C. T. (2014). Dithiocarbamates strongly inhibit the *β*-class carbonic anhydrases from Mycobacterium tuberculosis. *Journal of Enzyme Inhibition and Medicinal Chemistry*.

[150] Maresca A., Vullo D., Scozzafava A., Manole G., Supuran C. T. (2014). Inhibition of the *β*-class carbonic anhydrases from Mycobacterium tuberculosis with carboxylic acids. *Journal of Enzyme Inhibition and Medicinal Chemistry*.

[151] Kim J. K., Lomelino C. L., Avvaru B. S. (2018). Active-site solvent replenishment observed during human carbonic anhydrase II catalysis. *IUCrJ*.

[152] Chapman H. N., Fromme P., Barty A., White T. A., Kirian R. A., Aquila A. (2011). Femtosecond X-ray protein nanocrystallography. *Nature*.

[153] Lomelino C. L., Kim J. K., Lee C. (2018). Carbonic anhydrase II microcrystals suitable for XFEL studies. *Acta Crystallographica Section F: Structural Biology Communications*.

[154] Fischer M., Shoichet B. K., Fraser J. S. (2015). One crystal, two temperatures: cryocooling penalties alter ligand binding to transient protein sites. *ChemBioChem*.

[155] Keedy D. A., Kenner L. R., Warkentin M., Woldeyes R. A., Hopkins J. B., Thompson M. C. (2015). Mapping the conformational landscape of a dynamic enzyme by multitemperature and XFEL crystallography. *eLife*.

